# Synthesis of highly stable γ-Fe_2_O_3_ ferrofluid dispersed in liquid paraffin, motor oil and sunflower oil for heat transfer applications

**DOI:** 10.1039/c7ra13467c

**Published:** 2018-04-16

**Authors:** Mohd Imran, Aabid Hussain Shaik, Akhalakur Rahman Ansari, Abdul Aziz, Shahir Hussain, Ahmed Farag Fadil Abouatiaa, Afzal Khan, Mohammed Rehaan Chandan

**Affiliations:** Chemical Engineering Department, Faculty of Engineering, Jazan University P. O. Box. 706 Jazan 45142 Saudi Arabia; Chemical Engineering Department, School of Civil and Chemical Engineering, Vellore Institute of Technology Vellore TN 632014 India chandan1816@gmail.com; Centre of Nanotechnology, King Abdulaziz University Jeddah 21589 Saudi Arabia; Mechanical Engineering Department, Faculty of Engineering, Jazan University P. O. Box. 706 Jazan 45142 Saudi Arabia; Electrical Engineering Department, Faculty of Engineering, Jazan University P. O. Box. 706 Jazan 45142 Saudi Arabia; State Key Laboratory of Silicon Materials, School of Material Science and Engineering, Zhejiang University Hangzhou 310027 China

## Abstract

This article aims at the synthesis of highly stable γ-Fe_2_O_3_ magnetic nanoparticles and their ferrofluids using different base liquids such as liquid paraffin, motor oil and sunflower oil for heat transfer applications. Phase and morphology of the synthesized nanoparticles were probed using XRD, SEM and FTIR spectroscopy. The average nanoparticle size of γ-Fe_2_O_3_ magnetic nanoparticles was found to be 13 nm. Stability of the ferrofluids was monitored by visually observing the aggregation nature of the nanoparticles for 180 days. The ferrofluid prepared using motor oil as a base fluid exhibited high stability (for more than 1 year) and a mean enhancement of 77% in thermal conductivity at 1.5 vol% nanoparticles was observed as compared to base fluid. The viscosity of the ferrofluids was also measured and found to be 18, 38 and 8 cP at 27 °C for the liquid paraffin based, motor oil based and sunflower oil based ferrofluid, respectively.

## Abstract 2

Highly stable ferrofluid for greater enhancement of thermal conductivity.

## Graphical abstract

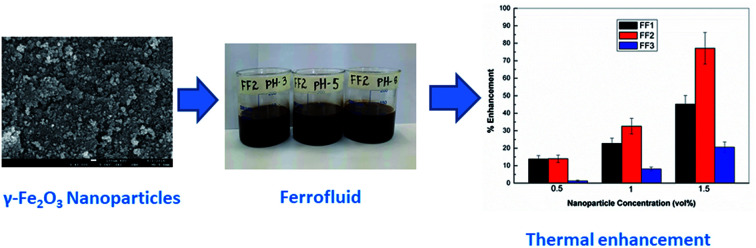

## 1. Introduction

Ferrofluids are colloidal dispersions of magnetic particles of size 10 nm in a liquid carrier. These fluids have drawn great interest in material research due to their distinct chemical, physical, mechanical and magnetic properties.^1^ The magnetic property of fluids helps in tracking the location and movement of ferrofluids and for precise control by applying and manipulating an external magnetic field.^2,3^ Ferrofluids find applications in technological, materials research and biomedical domains.^4,5^ Technological applications include dynamic sealing, heat dissipation, damping, doping of technological materials *etc.*^6^ In Biomedical field, ferrofluids are being used in cell separation, drug delivery in cancer therapy, magnetic induced hyperthermia, as a MRI contrast agent, immunomagnetic separation *etc.*^7,8^

Due to immense applications of ferrofluids in wide novel domains, researchers are focusing on synthesis of stable ferrofluids. Ferrofluids can be synthesized using nanoparticles of ferromagnetic metals as well as magnetic compounds. The most frequently used ferromagnetic metal is the iron. In specific, iron oxide especially Fe_2_O_3_ is the most common oxide of iron. This oxide of iron has been extensively focused in the current research community due to its magnetic and polymorphic properties. Among the polymorphs of Fe_2_O_3_*i.e.*, alpha, beta, gamma and epsilon,^9^ the stable structure and properties of alpha (hematite) and gamma (maghemite) are creating interest among researchers. Maghemite nanoparticles have been synthesizing using a variety of techniques which includes co-precipitation,^10^ sol–gel,^11^ microemulsion,^12^ ball-milling,^13^ and sonochemistry.^14^

Despite of synthesis of ferrofluids, stabilization of ferrofluids is a major challenging task. Stability of the ferrofluids depends on various parameters such as type of solvent, concentration of particles, pH *etc.* Bateer *et al.* (2014) synthesized Fe_3_O_4_ nanofluid using paraffin as a solvent and claim that nanofluid is highly stable in paraffin for 90 days.^15^ Jain *et al.* (2011) prepared ionic liquid ferrofluids containing bare and sterically stabilized superparamagnetic iron oxide nanoparticles dispersed in protic ethylammonium and aprotic imidazolium ionic liquids.^16^ Kim *et al.* (2005) synthesized oleic acid stabilized superparamagnetic iron oxide nanofluids using chitosan as a solvent.^14^ They observe that these nanofluids are stable for at least 30 days without showing any sign of aggregation. From the overview of the literature, it has been observed that the ferrofluids were mainly dispersed in ionic liquids, paraffin, chitosan solvents *etc.* to suit best for magnetic applications. However, the synthesis and application of stable ferrofluids using motor oil and sunflower oil as base solvents for heat transfer is very sparse in the literature other than a work recently published by our group on motor oil based ferrofluid (prepared using Fe_3_O_4_ nanoparticles) which shows enhancement of 51% in terms of thermal conductivity.^17^ This paper aims at resolving this problem.

Moreover, ferrofluids also shows good enhancement in thermal conductivity as compared to base solvents. The enhancement of thermal properties depends on various factors such as temperature, pH, volume fraction of nanoparticles *etc.*^18,19^ Yu *et al.* (2010) synthesized kerosene based Fe_3_O_4_ nanofluid using phase transfer method and reported that the thermal conductivity increases linearly with the volume fraction of Fe_3_O_4_ nanoparticles.^20^ Guo *et al.* (2010) prepared Fe_2_O_3_ nanofluid using mixture of ethylene glycol and water as base solvent and reported that these fluids are exhibiting greater enhancement in thermal conductivity and viscosity as compared to base solvents.^21^ Colla *et al.* (2012) synthesized water based Fe_2_O_3_ nanofluid and claims that the thermal conductivity and viscosity increases with increase in temperature and particle concentration.^22^

From the foregoing discussion, it has been observed that most of the work has been conducted on preparing moderately stable water based Fe_2_O_3_ nanofluids for heat transfer applications. No work has been conducted on preparing highly stable paraffin oil, motor oil and sunflower oil based Fe_2_O_3_ nanofluid for heat transfer applications. This paper focuses on the synthesis of highly stable Fe_2_O_3_ nanofluids (ferrofluids) using three different oils of varying viscosity such as paraffin oil, motor oil and sunflower oil as base fluids for heat transfer applications. Thorough analysis of stability of ferrofluids has been conducted by observing the aggregation behaviour of ferrofluids with respect to time. Thermal conductivity and viscosity measurements were also conducted by varying various parameters to enhance the thermal and rheological properties of base fluids for their application in heat transfer as coolants.

## 2. Experimental section

### 2.1 Materials

Ferrous sulfate heptahydrate (FeSO_4_·7H_2_O) and NaOH were purchased from Sigma-Aldrich Corporation, USA. Ferric chloride hexahydrate (FeCl_3_·6H_2_O) was procured from Scientific Limited, UK and extra pure oleic acid was obtained from Gem-Chem, India and used without further purification. The engine oil of 20W-50 grade and vegetable oil (sunflower) were purchased from SASO, Saudi Arabia. Paraffin oil was obtained from Loba Chemie, India. HCl was purchased from Chem-Lab, Belgium and absolute alcohol of analytical grade was purchased from Scharlau, Spain. De-ionized (D.I.) water used in this work was purified with a Puris-Expe water system.

### 2.2 Synthesis of γ-Fe_2_O_3_ nanoparticles

Iron oxide (γ-Fe_2_O_3_) nanoparticles were synthesized by a controlled co-precipitation technique. Initially, ferrous sulfate heptahydrate and ferric chloride hexahydrate were dissolved in deionised water. After that, the pH of the above solution was adjusted to 11 by adding alkaline solution drop by drop. After adding, the solution turned to black precipitate immediately and the resulting black precipitate was collected with a magnet and the supernatant was removed from the precipitate by decantation.^2^ Later, washing of the precipitate was conducted by treating the precipitate with deoxygenated ultrapure water and alcohol mixture. The procedure was repeated for 5 times. After washing the precipitate for 5 times, 0.01 M HCl solution was added to the precipitate to neutralize the anionic charges on the surface of nanoparticles. The resulting black powder was isolated using an external magnetic field. Finally, the magnetic nanoparticles were calcined in an oven at 450 °C for 3–4 hours.

### 2.3 Synthesis of iron oxide ferrofluids using paraffin oil, motor oil and sunflower oil as base fluids

Briefly, the synthesized magnetic nanoparticles (γ-Fe_2_O_3_) were initially coated with oleic acid by mixing 5% (w/v) Fe_2_O_3_ nanoparticles with 10% (v/v) oleic acid and the resultant viscous solution was stirred vigorously for 1 hour at 40 °C. After stirring, the viscous solution was transferred into a beaker and diluted to 100 ml by adding liquid paraffin oil to obtain colloidal solution. This colloidal solution was further ultra-sonicated for 1 h at 70 °C to obtain stable nanofluid. After sonication, the prepared nanofluid was kept in a bottle and the stability of the ferrofluid was observed with respect to time. The above procedure has been repeated for the preparation of ferrofluids using motor oil and sunflower oil as base solvents.

### 2.4 Measurement of thermal conductivity

Heat transfer studies were conducted in WL-373 heat conduction instrument which is particularly calibrated for the estimation of thermal conductivity of liquids and gases. Generally, this unit comprises a double walled cylinder with an integrated heater acting as a heat source, and the surrounding cylinder as a heat sink. The medium to be investigated is placed in between a measurement slot. After that, the temperature of heat source and sink were measured using thermocouples and transmitted to a measurement control unit. The heat transfer in the medium is completely due to thermal conduction. Due to the constant width of the measurement slot, it has been assumed that thermal conduction occurs in a plane wall. Hence, the rate of heat flow is calculated using Fourier law:
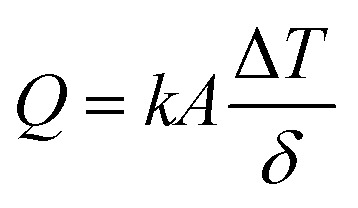


### 2.5 Characterization

The morphology of nanoparticles was analyzed using Scanning Electron Microscope (JEOL JSM-7600F). The phase purity of nanoparticles were investigated using X-ray diffraction technique (XRD) with D8 AαS Advance X-ray diffractometer using Cu Kα radiation (*λ* = 1.54156 Å). The surface bonding was measured using Fourier Transform Infrared (FTIR) spectroscopy (ATR-FT-IR model ‘‘Nicolet IS 10’’) equipped with the specular reflectance accessory. The viscosity of ferrofluid was investigated using a viscometer (Brookfield DV-II + Pro). The thermal conductivity of ferrofluids was estimated using WL-373 heat conduction instrument (GUNT, Germany).

## 3. Result and discussion

### 3.1 Phase and surface morphology

**Fig. 1:**
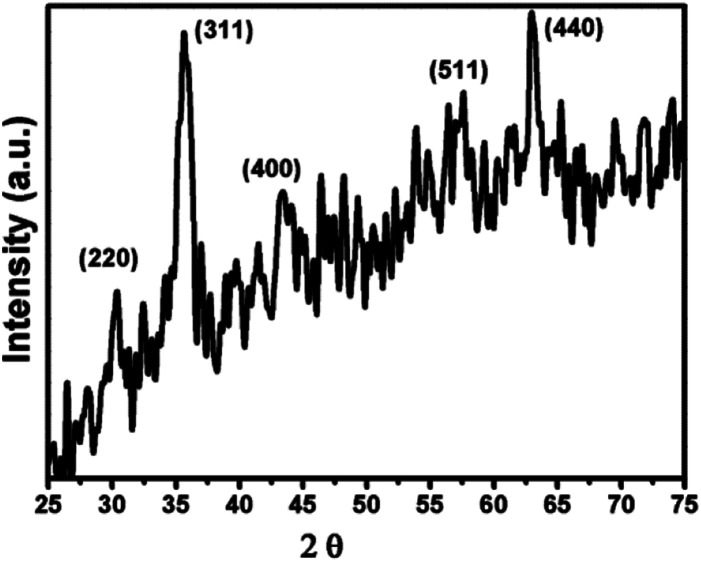
XRD of Fe_2_O_3_ nanoparticles.

Three different types of ferrofluids have been prepared using different base fluids. Ferrofluid-1 (FF-1) was prepared by dispersing γ-Fe_2_O_3_ nanoparticles in paraffin oil. Similarly, ferrofluid-2 (FF-2) and ferrofluid-3 (FF-3) were prepared using motor oil and sunflower oil as base fluids. Fig. 1 represents the XRD of γ-Fe_2_O_3_ nanoparticles synthesized using controlled co-precipitation technique. The peaks situated at several diffraction angles such as 31, 36, 43.5, 57 and 63.5 suggests 220, 311, 400, 511 and 440 crystalline phases of γ-Fe_2_O_3_ nanoparticles (PDF no.-01-089-5892). Moreover, the increased resolution of the planes with peak broadening suggests that γ-Fe_2_O_3_ crystal was showing a mean size of 12.6 nm.

**Fig. 2:**
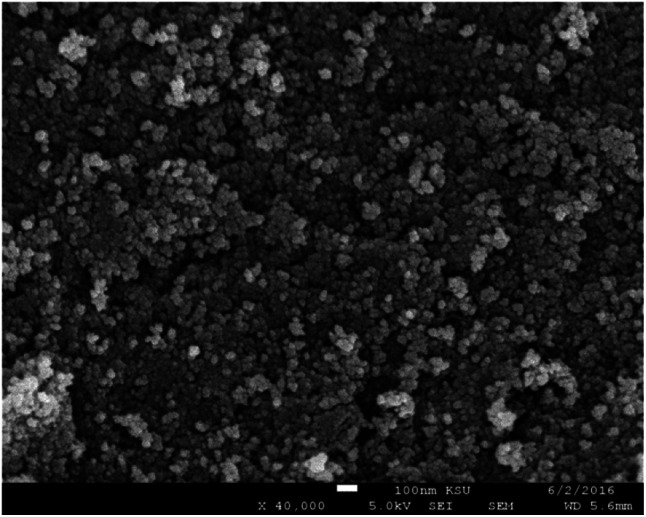
SEM image of γ-Fe_2_O_3_ nanoparticles.

**Fig. 3:**
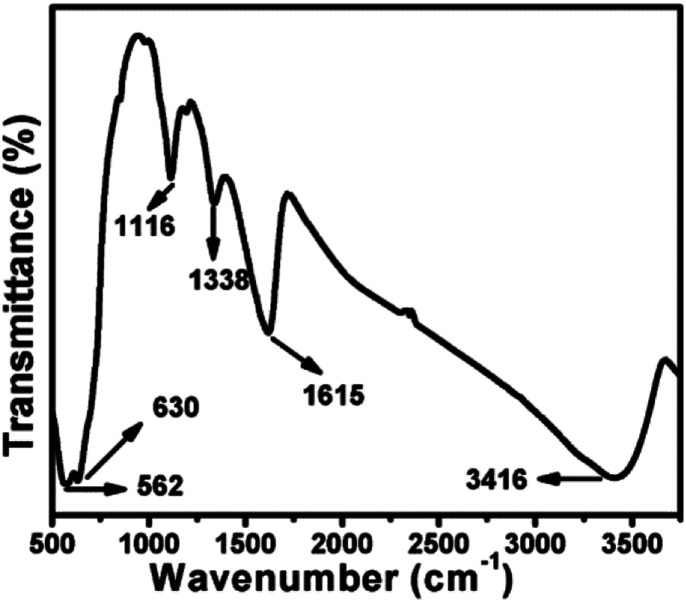
FTIR spectra of γ-Fe_2_O_3_ magnetic nanoparticles.

Fig. 2 shows the SEM of γ-Fe_2_O_3_ nanoparticles. The particles are showing slight agglomerating nature for high concentration of particles even though the sample was sonicated for 15 minutes in motor oil before drop casting the sol on the aluminium foil. From the figure, it has been observed that the particles are spherical in nature and showing a mean size of 13 nm (around 100 particles were taken from Fig. 2 for calculating mean size using ImageJ software) which is in consistent with the crystallite size calculated from the XRD analysis. The aggregation behavior of the particle were possibly due to the enhanced magnetic and inter particle interaction between the particles.^2^ The nature of produced ferrofluid was observed using FTIR analysis. Fig. 3 shows the FTIR spectra of γ-Fe_2_O_3_ nanoparticles synthesized by co-precipitation method. The sample for FTIR analysis was prepared as a pellet by mixing γ-Fe_2_O_3_ nanoparticles with KBr powder using hydraulic press. The characteristic bands at 562 cm^−1^ and 630 cm^−1^ were attributed to Fe–O bonding in γ-Fe_2_O_3_ nanoparticles. Another characteristic peak has been noticed at 3416 cm^−1^ corresponds to –OH group of water. A small dip in the FTIR spectra has been observed at 2362 cm^−1^ due to the presence of atmospheric carbon dioxide.^23^

### 3.2 Stability of ferrofluids

**Fig. 4:**
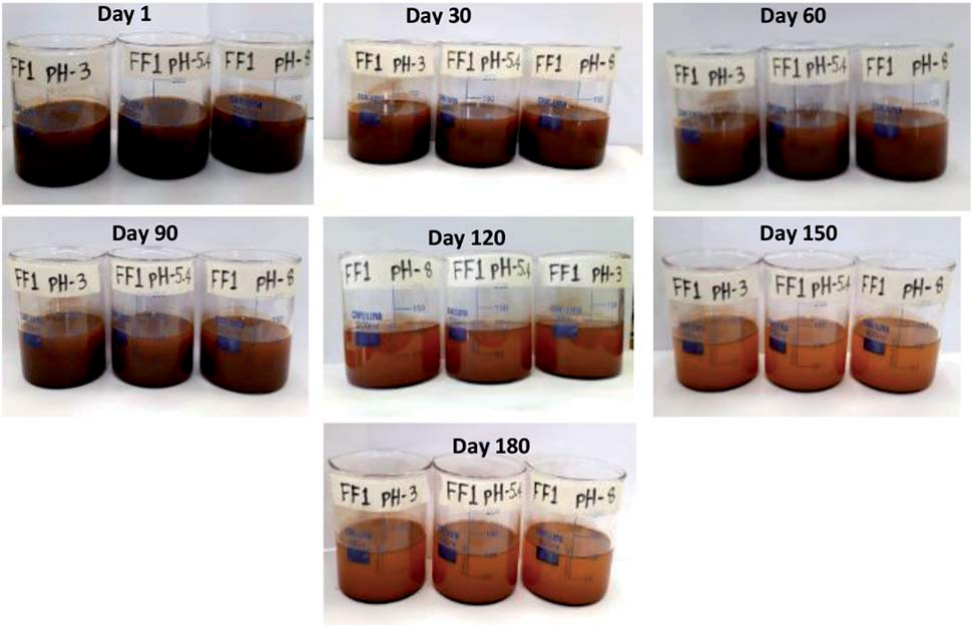
Digital photo images of FF-1 with respect to time.

**Fig. 5:**
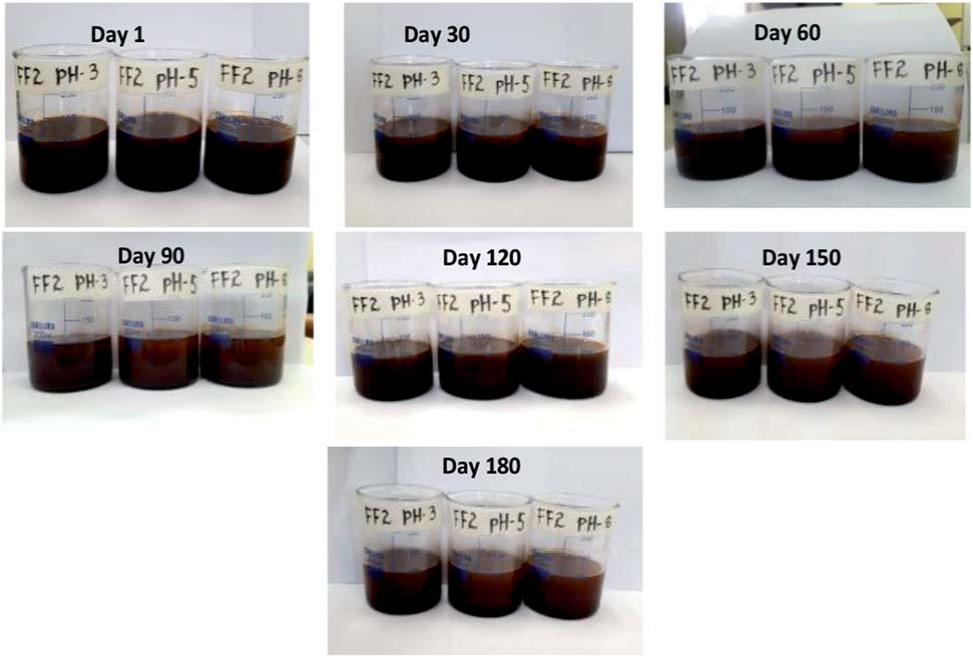
Digital photo images of FF-2 with respect to time.

**Fig. 6:**
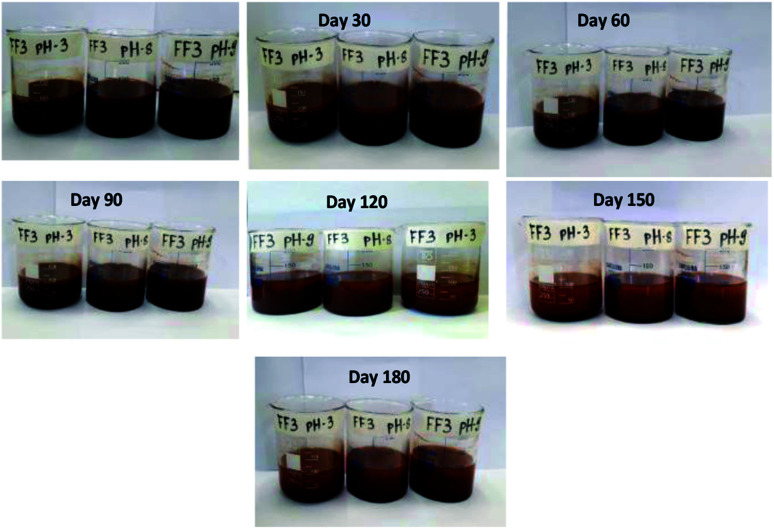
Digital photo images of FF-3 with respect to time.

Since stability is the most important characteristics of ferrofluid, the detailed stability analysis of prepared ferrofluids was conducted by observing the aggregation behavior of ferrofluids with respect to time. In this work, the stability analysis of 1.5 vol% nanoparticle based three ferrofluids (FF-1, FF-2 and FF-3) was conducted by storing the ferrofluids in a beaker in an open ambience for 180 days. Digital images of the ferrofluids were taken in regular intervals of time to observe the aggregation behavior of nanoparticles in fluid. The digital images of ferrofluids with respect to time were shown in Fig. 4 (FF-1), Fig. 5 (FF-2) and Fig. 6 (FF-3) respectively.

Fig. 4 represents the stability analysis of FF-1 synthesized using paraffin oil as a base fluid. From the figure, it has been observed that ferrofluid is highly stable for 30 days and partially stable for 120 days. After 120 days, slight formation of flocs was noticed in the beaker due to detachment of oleic acid from the surface of nanoparticles. Complete aggregation of nanoparticle was not observed even after 180 days. These formed flocs were broken down by mild sonication for 5 minutes. After sonication, ferrofluid is showing good stability *i.e.* stable for more than 1 month.

Fig. 5 show the stability of FF-2 with respect to time. FF-2 is showing highly stable nature even after storing for 180 days in open atmosphere.

No sign of aggregation has been noticed in the beaker even after 180 days. From the figure, it is clear that even flocs are not formed. This high stability of ferrofluid in motor oil may be due to the refractive index or dielectric constant of the solvent.^24–26^ Formation of flocs has been observed after 9 months and these flocs can be broken down by mild sonication. After sonication, ferrofluid is highly stable for more than one year.

Fig. 6 represents the visual observation of aggregation behaviour in FF-3. From the figure, it can be seen that the ferrofluid is highly stable for 90 days and partially stable for 120 days. After 120 days, a slight formation of flocs was observed which can be broken by mild sonication for 5 minutes.

### 3.3 Thermal properties of ferrofluids

**Fig. 7:**
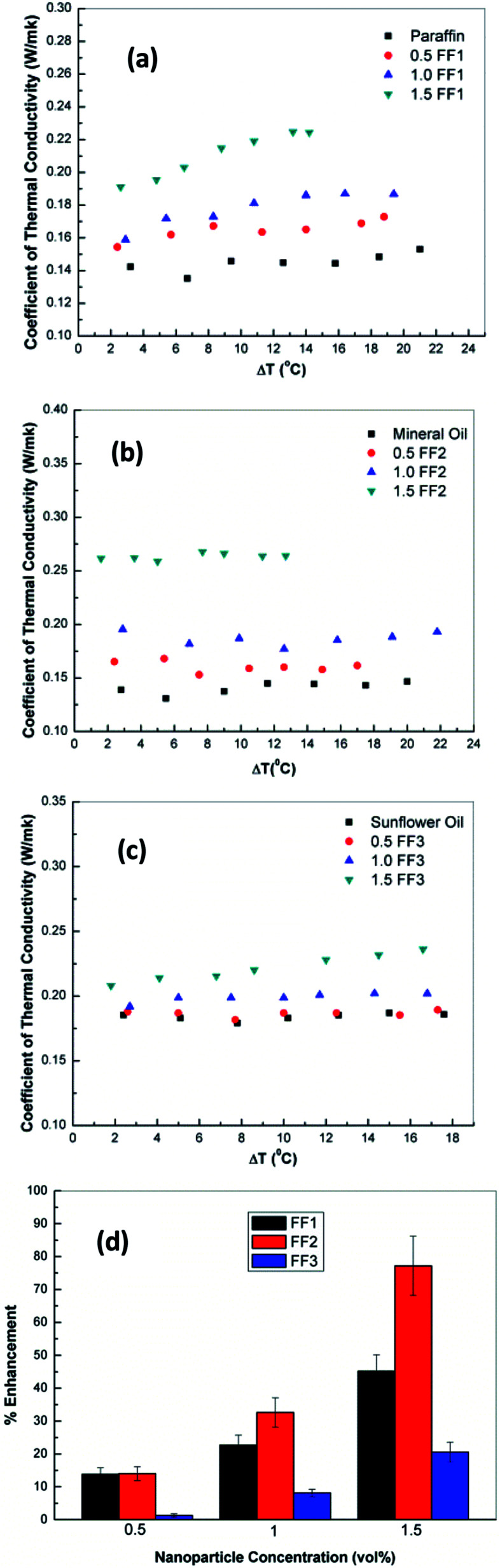
Variation of co-efficient of thermal conduction as a function of γ-Fe_2_O_3_ nanoparticles concentration in (a) liquid paraffin (b) motor (c) sunflower oil base fluid. (d) Variation of % enhancement of thermal conductivity as a function of γ-Fe_2_O_3_ nanoparticles concentration.

The variation of coefficient of thermal conduction for FF1 with various volume fraction of γ-Fe_2_O_3_ nanoparticles (0.5, 1.0 and 1.5 vol%) is shown in Fig. 7(a). From figure, it can be seen that the thermal conductivity of FF-1 increases with increase in the concentration of γ-Fe_2_O_3_ nanoparticles. The values of thermal conductivity at 0.5, 1.0 and 1.5 vol% are 0.145, 0.165 and 0.210 W mK^−1^ respectively. FF-1 prepared by using 1.5 vol% of γ-Fe_2_O_3_ nanoparticles shows 45.2% enhancement (mean) of thermal conductivity as compared to paraffin oil. Similar trends were observed for FF-2 and FF-3 as shown in Fig. 7(b) and (c). The mean enhancement of thermal conductivity in FF-2 and FF-3 are 77% and 20.6% as compared to mineral oil and sunflower oil as shown in Fig. 7(d). A remarkable enhancement of thermal conductivity was observed in ferrofluids prepared using mineral oil as base fluid due to highly stable nature of ferrofluid, small size of nanoparticle and interaction between γ-Fe_2_O_3_ nanoparticles and the basefluid.^27^

### 3.4 Rheological properties of ferrofluids

**Fig. 8:**
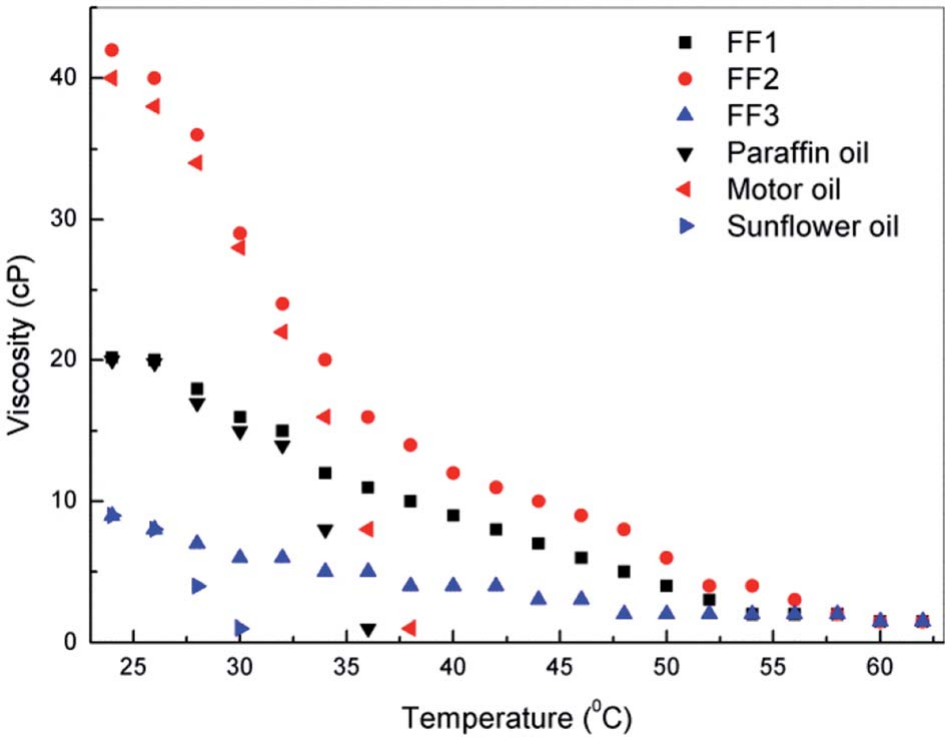
Variation of viscosities of 1.5 vol% nanoparticle loaded ferrofluids (FF-1, FF-2 and FF-3) with respect to temperature.

Rheological behavior of ferrofluids significantly impacts their application in various fields such as in valves, pneumatic servo controller, and shock absorber *etc.*^28^ Viscosity is one of the important properties associated with ferrofluid, which depends on the nature of carrier liquid as well as the concentration and size of magnetic nanoparticles.^29^ The viscosity of ferrofluids for 1.5 vol% nanoparticle concentration were measured initially at room temperature (*i.e.*, 27 °C) and found to be 20 cP, 42 cP and 8 cP for FF-1, FF-2 and FF-3 respectively. These viscosities of ferrofluids were compared with the base fluids as shown in the Fig. 8. Viscosities of all ferrofluids and base fluids were found to be decreasing with temperature and for all ferrofluids it was found to be around 1 cP at 55 °C. Moreover, viscosities of base fluids are comparable with their ferrofluid counterpart till 33 °C for both paraffin and motor oil, whereas 28 °C for sunflower oil. This confirms that the synthesized ferrofluids is influenced by the presence of nanoparticles at higher temperatures as reported by Ahammed *et al.* (2016).^30^

## 4. Conclusions

γ-Fe_2_O_3_ nanoparticles was successfully synthesized by co-precipitation method and its ferrofluids were prepared by dispersing nanoparticle in paraffin oil, motor oil and sunflower oil. The average crystal size of γ-Fe_2_O_3_ nanoparticles was calculated (12.6 nm) using Scherrer equation from XRD analysis. SEM analysis confirms the spherical nature of the particles and average size was also comparable with the XRD analysis result. A remarkable enhancement (mean) in thermal conductivity was observed for motor oil based ferrofluid (FF-2) *i.e.*, 77%. Viscosity behavior of ferrofluids was found to be similar for all ferrofluids with increase in temperature. This confirms the high heat transfer rate and flow ability of ferrofluids in heat transfer applications such as in coolant pipes and other channels.

## Conflicts of interest

There are no conflicts of interest with the authors.

## Supplementary Material
